# An Intergenic Non-Coding rRNA Correlated with Expression of the *rRNA* and Frequency of an *rRNA* Single Nucleotide Polymorphism in Lung Cancer Cells

**DOI:** 10.1371/journal.pone.0007505

**Published:** 2009-10-19

**Authors:** Yih-Horng Shiao, Sorin T. Lupascu, Yuhan D. Gu, Wojciech Kasprzak, Christopher J. Hwang, Janet R. Fields, Robert M. Leighty, Octavio Quiñones, Bruce A. Shapiro, W. Gregory Alvord, Lucy M. Anderson

**Affiliations:** 1 Laboratory of Comparative Carcinogenesis, National Cancer Institute at Frederick, Frederick, Maryland, United States of America; 2 Basic Science Program, Science Applications International Corporation-Frederick Incorporate, Frederick, Maryland, United States of America; 3 Data Management Services Incorporate, Frederick, Maryland, United States of America; 4 Center for Cancer Research Nanobiology Program, National Cancer Institute at Frederick, Frederick, Maryland, United States of America; Innsbruck Medical University, Austria

## Abstract

**Background:**

Ribosomal RNA (rRNA) is a central regulator of cell growth and may control cancer development. A cis noncoding rRNA (nc-rRNA) upstream from the *45S rRNA* transcription start site has recently been implicated in control of *rRNA* transcription in mouse fibroblasts. We investigated whether a similar nc-rRNA might be expressed in human cancer epithelial cells, and related to any genomic characteristics.

**Methodology/Principal Findings:**

Using quantitative rRNA measurement, we demonstrated that a nc-rRNA is transcribed in human lung epithelial and lung cancer cells, starting from approximately −1000 nucleotides upstream of the *rRNA* transcription start site (+1) and extending at least to +203. This nc-rRNA was significantly more abundant in the majority of lung cancer cell lines, relative to a nontransformed lung epithelial cell line. Its abundance correlated negatively with total 45S rRNA in 12 of 13 cell lines (P = 0.014). During sequence analysis from −388 to +306, we observed diverse, frequent intercopy single nucleotide polymorphisms (SNPs) in *rRNA*, with a frequency greater than predicted by chance at 12 sites. A SNP at +139 (U/C) in the 5′ leader sequence varied among the cell lines and correlated negatively with level of the nc-rRNA (P = 0.014). Modelling of the secondary structure of the rRNA 5′-leader sequence indicated a small increase in structural stability due to the +139 U/C SNP and a minor shift in local configuration occurrences.

**Conclusions/Significance:**

The results demonstrate occurrence of a sense nc-rRNA in human lung epithelial and cancer cells, and imply a role in regulation of the *rRNA* gene, which may be affected by a +139 SNP in the 5′ leader sequence of the primary rRNA transcript.

## Introduction

Synthesis of ribosomes demands a high proportion of cellular resources and hence is highly regulated. Biogenesis of the 45S ribosomal RNA (rRNA) is rate-limiting for ribosome synthesis and is closely controlled at multiple levels. An increase in ribosomes is a common feature of actively proliferating cells, including cancer cells, may be inducible by oncogene activation or inactivation of tumor suppressors, and may even be cancer-causative [1], [2].

A new type of rRNA regulator has recently been discovered in mouse fibroblasts: a sense non-coding RNA (nc-rRNA) originating from the intergenic spacer region between *rRNA* tandemly repeated genes.It was shown to function as a negative regulator of rRNA expression [3], [4]. The possible occurrence and significance of this nc-rRNA in human cells, in cells of epithelial origin, and in cancer cells have not been studied. We investigated whether this nc-rRNA might be found in human lung epithelial cells and in human lung cancer cells. We also noted the occurrence of single nucleotide polymorphisms (SNPs) in the *rRNA* gene among the cell lines, in the sequences upstream and downstream of the *rRNA* transcription start site, and studied their relationships to the nc-rRNA levels and to potential folding of the rRNA.

## Results

### Single nucleotide polymorphisms in *rRNA* near the transcription start site

**Figure 1:**
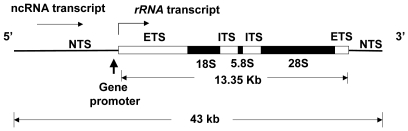
Repeat unit of the *45S rRNA* gene (tandemly repeated in human chr. 13, 14, 15, 21, and 22). NTS: non-transcribed spacer, also known as the intergenic region; ETS: external transcribed spacer; ITS: internal transcribed spacer. rRNA transcription starts at the 5′ end of the ETS, with the first 414 nucleotides comprising the leader sequence. The non-coding (nc) r-RNA transcript is initiated upstream of the ETS.

In humans, some 400 *rRNA* gene copies are arranged in sets of tandem repeats on 5 chromosomes [5]. The arrangement of the 18S, 5.8S, and 28S *rRNA* genes is diagrammed in Fig. 1. Their products are processed from a 45S precursor rRNA, which begins with a 5′-external transcribed sequence; the first 414 nucleotides of this constitute a leader sequence and is the first part to be removed [6]. The core promoter is located at approximately −45 to +18 relative to the transcription start site. There are also regulatory enhancer elements in the intergenic spacers [reviewed in 7].

Six human lung adenocarcinoma cell lines and a nontransformed immortalized line from human peripheral lung epithelium were studied at the genomic level. For each cell line, 35–65 clones of amplified products for the region −388 to +306 (relative to the +1 transcription start site) were obtained and sequenced. In comparisons with sequences for an *rRNA* from chromosome 22 (GenBank AL592188), among the seven cell lines there was at least one SNP at 329 of the 694 sites (47.4%). Detailed data are given in Supplementary Table S1. At least one SNP was found in 68% to 90% of the clones from the various cell lines (Table 1). The percentages were not significantly different among the lines. Among the SNP-positive clones, the average numbers of SNPs was 2.1 to 3.1, with no significance differences among the cell lines. The percentages of clones stratified by numbers of SNPs also did not vary significantly across the cell lines (Table 1).

**Table 1:** *rRNA* SNPs in HPL non-cancer and 6 lung cancer cell lines.

Cell line	Clones with SNP/total (%)	Av # SNPs±S.E.	% clones with 1–2, 3–5, or 6+ SNPs			Multiple clones with same SNPs	Hotspot SNPs^c^
			1–2	3–5	6 or more		
All lines	282/362 (77.9)	2.5±0.3	68	27	5		−234 (9), −233 (10), −181 (6), −104 (6), −96 (28), −72 (6), +52 (7), +139 (51), + 144 (6), +207 (9), +225 (7), +290 (6)
HPL	38/51 (74.5)	2.3±0.2	71	26	3	+30 & +52 (2)^a^ +21 & +287 (2)	−234 (4), −233 (2), −181 (3), +30 (4), +52 (5), +139 (4)
A549	47/54 (87.0)	2.6±0.2	53	45	2	−96 & +139 (7)^b^ −96, +139, & −62 (2)	−234, −233 (2), −104 (5), −96 (22), +139 (28), +144 (2), +225
H441	29/36 (80.6)	2.3±0.4	76	17	7	+180 (3), +298 (2)	−233, −234, +139 (7), +144, +225
H23	43/48 (89.6)	2.5±0.3	70	23	7	+139 (3), +139 & +213 (2)	−234 (2), −233 (2), +139 (7), +207 (9), +225 (3)
H1792	41/52 (78.8)	3.1±0.6	63	27	10	−216 & −40 (2), −182 & −76 (2)	−234, −233, −96 (2), −72, +52, +290
H2030	40/56 (71.4)	2.1±0.3	73	22	5	−110 (2), −73 (2), +290 (3)	−233, −181 (3), −96 (4), −72 (2), +52, +139 (3), +144 (3), +290 (3)
H2122	44/65 (67.7)	2.4±0.2	68	27	5	None	−233, −104, −72 (3), +139 (2), +225 (2), +290 (2)

a 2 additional clones had these SNPs plus 3 or 4 others.

b 13 additional clones had these SNPs plus 1 to 4 others.

c Occurrence in each cell line of clones with SNPs that have significant incidence in that cell line, or in all lines considered together. Multiple clones are indicated in parentheses. Underlined sites are those with a higher number of clones than expected by chance (N≥4), in that cell line.

We determined whether individual sites were more likely to present a SNP than expected by chance, for all seven cell lines considered together. Probabilities obtained from the Poisson distribution indicated that occurrence of SNPs in 6 or more clones at a given site was highly unlikely (P<0.0001) by chance. There were 12 such sites (total number of clones in parentheses): −234 (9), −233 (10), −181 (6), −104 (6), −96 (28), −72 (6), +52 (7), +139 (51), +144 (6), +207 (9), +225 (7), and +290 (6). The occurrences of these hotspot SNPs in the cell lines are shown in Table 1. Of these, several are especially notable. Three different SNPs were found at site −233 (T/A, T/C, or deletion, Supplementary Table 1), with at least one SNP in each cell line. Line A549 had noticeably more SNPs at −96 (22 SNPs) and +139 (28 SNPs), compared with the other cells lines (Table 1). SNP +139 in particular presented high frequency in several lines.

We examined statistically whether there were sequence stretches where the frequency of SNPs was higher or lower than predicted by chance. The runs test did not reveal any such stretches for cell lines A549, H23, H1792, H2030, H2122, and HPL1D and for all of the lines considered together. However for line H441 considered separately, the absence of SNPs for the regions −232 to −180, and −94 to −26, was not expected by chance alone (P = 0.0072). Several cell lines appeared to exhibit a high frequency of SNPs in the region −239 to −228. The entire region was partitioned into two strata, a potential SNP hotspot stratum from −239 to −228, and the complementary stratum consisting of the remaining sites. Poisson regression (with an offset parameter equalling the number of sites) was used to compare the rates of SNPs in these two strata. The SNP rate was 3.7 times greater in the potential SNP hotspot stratum than elsewhere, P = 0.0010. The rate increases were especially prominent for the H1792, HPL1D, and H23 lines: 3.9, 7.3, and 9.0 times greater respectively (all P values≤0.0002).

For most cell lines there were several instances of more than one clone presenting the same SNP(s), in most cases 2 or 3 clones. It cannot be decided with certainty if these represent separate alleles, or multiple polymerase chain reaction (PCR) products from the same allele. There were 1 to 3 such duplicate pairs in each cell line, in addition to seven clones in A549 containing both −96 and +139 SNPs (Table 1). SNP −96 was found only in clones with the +139 SNP in A549 cells. In addition, there were many instances of clones with one to three identical SNPs, plus one to many additional SNPs unique to each clone. These clones obviously represent different alleles. In each cell line there were 9 to 15 SNP sites involved in such combinations, except for H441 cells where only site +139 was found in seven such variant clones. Aside from the association of SNP −96 with SNP +139 in *rRNA* clones from A549 cells, multiple different SNPs appeared to occur at random in the clones from this and the other cell lines.

In view of the frequency of SNP +139 and the differences among the cell lines, we examined its occurrence by an additional technique. RNA was isolated from the seven cell lines listed in Table 1, plus seven additional lung adenocarcinoma cell lines (Table 2). The sequence surrounding position +139 was amplified by PCR after reverse transcription (RT) and the frequency of the two alleles determined quantitatively by pyrosequencing. The added benefits of this approach included evaluation of expression of the alleles as rRNA, and assessment of the whole population of molecules, whereas genomic cloning examined only a random sample. The results (Table 2) showed good agreement between the two methods. The correlation between frequency of the +139 SNP in genomic *rRNA* (ascertained by cloning) vs that in rRNA (determined by RT-PCR and pyrosequencing) is significant (P = 0.0073), suggesting that both alleles were equally expressed. Where there were differences in frequency, e.g. A549 cells, this may be a result of less accuracy in the estimation of the frequency by the cloning assay.

**Table 2:** Frequency of SNP +139 by cloning and by pyrosequencing methods.

Cells	Number clones (percent of all clones) with genomic SNP +139	Percent SNP +139 in rRNA by pyrosequencing method
HPL	4 (7.8)	8.2
A549	28 (51.8)	25.2
H441	7 (19.4)	18.7
H23	7 (14.6)	8.5
H1792	0	7.8
H2030	3 (5.4)	5.2
H2122	2 (3.1)	11.4
H727	Not done	12.2
H1944	Not done	20.4
H1395	Not done	10.4
H2126	Not done	20.3
H2023	Not done	10.3
H1703	Not done	45.3
H1355	Not done	21.3

### A non-coding RNA transcribed from *rRNA* upstream of the transcription start site

**Figure 2:**
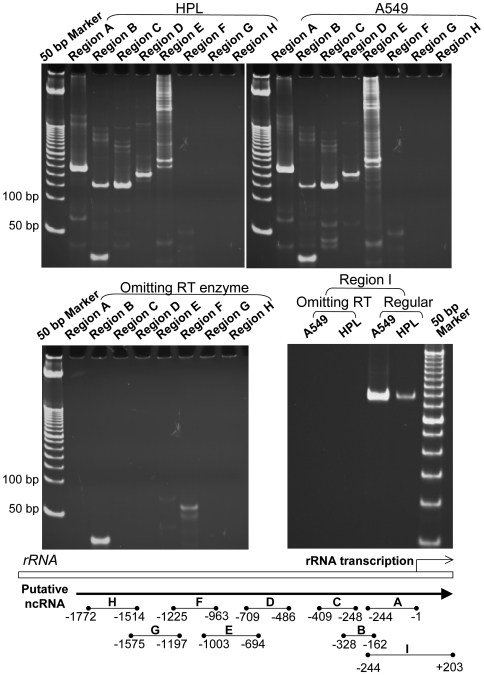
RT-PCR products corresponding to nc-rRNA of the human *rRNA* gene promoter and upstream sequences. The products were also confirmed by DNA sequencing. Representative findings are shown for cell lines HPL (immortalized non-transformed cells from human peripheral lung epithelium) and A549, a human lung adenocarcinoma cell line. Results for controls omitting the RT enzyme are shown in the lower panels. The positions amplified by specific primers are illustrated at the bottom of the figure, with +1 being the transcription start site for rRNA. The nc-rRNA was detected between −1003 and +203, but not between −1772 and −1225.

An nc-rRNA from the *rRNA* intergenic spacer was recently described for mouse fibroblasts [3], [4], but the extent of the original transcript was not completely defined. Using primers for the region −244 to +203 relative to the human *rRNA* transcription start site, we demonstrated that an nc-rRNA for this region was transcribed by human epithelial cells also, including both the nontransformed HPL1D cells and lung adenocarcinoma A549 cells (Fig. 2, region I). The transcript of the nc-rRNA was confirmed by sequencing. To determine the upstream start site for this nc-rRNA, several primers were utilized, starting at −1772. Sequencing-confirmed products were obtained with primers A–E (−1003 to −1), whereas primers F, G, and H, covering more upstream sequences, showed no products (Fig. 2). Therefore transcription of the nc-rRNA commenced between −1225 and −1003.

**Figure 3:**
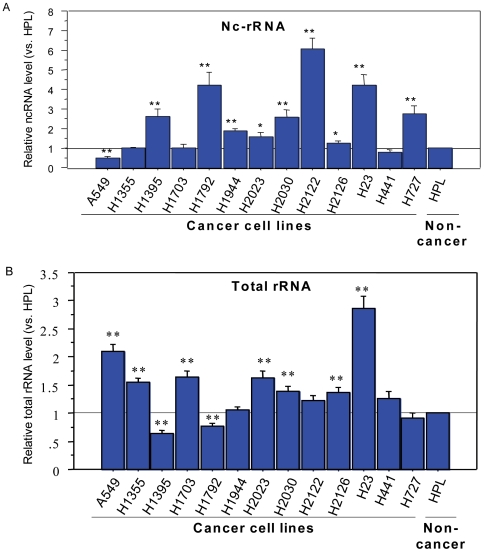
Average levels of nc-rRNA from 7 RT-PCR repeats (A) and 45S rRNAs from 9 repeats (B) in a series of transformed human lung cancer cell lines compared to the non-cancerous HPL cell line. * P<0.05, ** P<0.01, significantly different from non-transformed HPL cells. For 45S rRNA, apparent increases were of borderline significance for H2122 (p = 0.062) and for H441 (P = 0.0595).

The amounts of this nc-rRNA varied in the lung cell lines, with nine adenocarcinoma cell lines exhibiting significantly more than the nontransformed line HPL1D. One line, A549, had significantly less nc-rRNA, and the levels were not significantly different in three lines (Fig. 3A).

**Figure 4:**
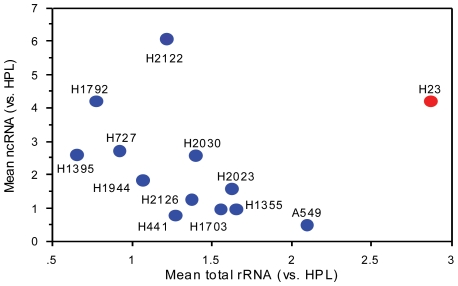
Correlation of averages levels of total 45S rRNA (relative to HPL cells) with average levels of nc-rRNA (relative to HPL cells) in human lung cancer lines. When outlier line H23 was omitted, the Spearman rank correlation coefficient rho = −0.74, P = 0.0135 by the Spearman rank test.

Since in the mouse fibroblast cells the *rRNA* intergenic spacer ncRNA had a negative regulatory effect on rRNA [3], [4], we quantified total rRNA with real-time PCR after RT. Relative to HPL1D, seven cancer lines expressed significantly more rRNA, two significantly less, and four were not significantly different (Fig. 3B). For twelve of the thirteen lung cancer cell lines, there was a significant negative rank correlation between levels of ncRNA and total rRNA transcript (Fig. 4). The outlier cell line, H23, had the highest levels of total rRNA. A provisional conclusion, for future exploration, is that the nc-rRNA and rRNA are functionally linked in the majority of these lung cancer cells. By analogy to their relationship in mouse fibroblasts [3], [4], the nc-rRNA may be a negative regulator of the rRNA. In the H23 outlier, other factors may have added importance in rRNA regulation.

### Site +139 SNP correlated inversely with nc-rRNA

**Figure 5:**
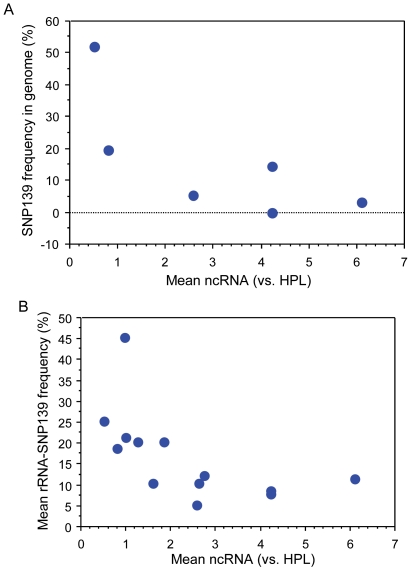
Correlation of average levels of nc-rRNA (relative to HPL cells) with frequency of the SNP at +139. A. Correlation of the genomic SNP determined by cloning in 6 human lung cancer cell lines, A549, H441, H23, H1792, H2030, and H2122 (see Table 2) (Spearman rank correlation coefficient, rho = −0.89, P = 0.0409, by the Spearman rank test). B. Correlation of the SNP as variant *rRNA* transcript in 13 cancer lines (Spearman rank correlation coefficient, rho = −0.71, P = 0.0137, by the Spearman rank test).

We next inquired whether SNP +139, that occurred in most of the cell lines and varied significantly among them, had a relationship to nc-rRNA and total rRNA expression. The frequency of the T to C genomic SNP at position +139 in the original panel of six cancer cell lines had a significant negative rank correlation with nc-rRNA (Fig. 5A). This association was investigated in more detail by use of the whole panel of cell lines and the determination of SNP allele frequencies in the expressed rRNA by pyrosequencing (see Table 2). With the rRNA data for all of the cell lines, there was again a significant negative rank correlation between SNP +139 frequency and nc-rRNA levels (Fig. 5B).

### Secondary structure models of rRNA species

**Figure 6:**
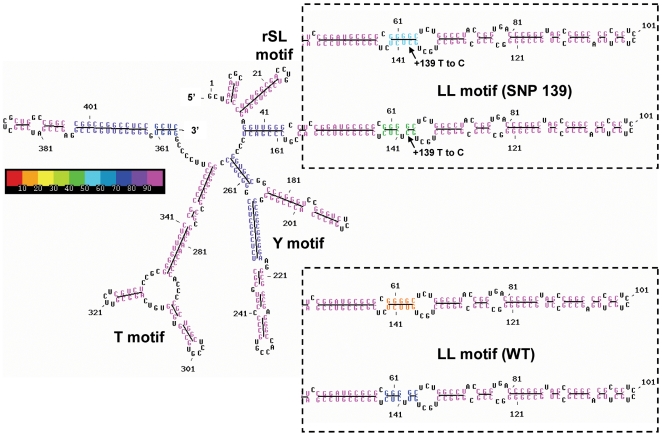
Dominant secondary structures predicted by MPGAfold for the +1 to +414 5′-leader sequence of 45S rRNA. The stems are color-coded (scale shown) based on their frequency of occurrence in the final structure among 20 runs at the population level of 128 K. Unpaired bases are black. The basic structure at left was highly conserved and was as shown in all but 4/40 of the final predictions. The dashed boxes at right showed alternative conformations of the LL motif surrounding the +139 site, which differ for the 5 nucleotides at 60–64 and 138–142. For the wild-type, +139 U (bottom dashed box), the conformation of these nucleotides involved predominately a 3-nucleotide stem and a looped-out U at +140 (dark blue in the diagram). This LL motif variant was found in 80% (16/20) of the final structures, with E = −246.1 kcal/mol in all but one of these. The LL motif structures with the 5 nucleotides in orange, all as part of the stem, were in the minority, 20% (4/20) of all solutions (see Table 3). The full structure illustrated was identical for all but one of these, with E = −245.9 kcal/mol. By contrast, for the SNP +139 C (top dashed box) the two conformations were presented in similar proportions, 45% as the 3-nucleotide stem (green), E = −248.8 kcal/mol, and 55% as the 5-nucleotide stem (light blue), E = −248.4 kcal/mol.

Stable secondary structure has been reported previously for the 5′-leader sequence of rRNA [8], [9]. We considered whether the +139 SNP might alter the secondary structure, and hence the potential functionality, of the rRNA transcript or of the nc-rRNA. We modelled co-transcriptional folding of the leader sequence, +1 to +414, using MPGAfold, including either U or C at site +139. There were 5 major stem structures, which we have designated as shown in Fig. 6. This overall structure was stable and conserved, giving the same base-pairing pattern in 70 to 100% of runs, as illustrated by the blue or magenta color coding in Fig. 6. The +139 site is located in a long stem structure, termed the LL motif, made up of sequences including +46 to +152 (extending as far as +38 to +166 in some modelling outcomes). The characteristics of the LL motif with U or C at site +139 are compared in Table 3. The SNP form +139 was consistently, if slightly, more stable, with a −2.7 kcal/mol advantage in both best free energy and dominant free energy in the LL folds. Two common local variants of the LL motif exist, one in which the +139 site occurs in a 5-nucleotide stem next to a bulge, and another where an unpaired +140 U next to +139 loops out (Fig. 6), for a 3-loop-2 stem configuration. As the MPGAfold population levels increased, the frequency of the better fit final structures containing the 3-loop-2 stem within the LL motif also increased, at the expense of the less fit 5-base pair stem (Table 3). This trend was noted for both wild type +139 U and SNP +139 C, but the latter retained a higher percentage of the 5-nucleotide conformation. The free energies of both conformations of +139 C were slightly more favourable than those of the +139 U conformations. This may well be related to pairing of +139 C to the G at +63.

**Table 3:** Comparison of co-transcriptional models of the LL motif rRNA leader sequence (+1 to +414) with C vs U at SNP site +139.

	Wild-type (+139 U)				SNP (+139 C)			
Population size modelled	% LL motif in conformation listed		Free Energy (kcal/mol)		% LL motif in conformation listed		Free energy (kcal/mol)	
	5 nt stem	3 nt stem	Best	Dominant	5 nt stem	3 nt stem	Best	Dominant
16 K^a^, 25 runs	92%	4%	−245.9	−245.9	84%	12%	−248.8	−248.4
64 K, 25 runs	44%	56%	−246.3	−246.1	80%	20%	−249.0	−248.8
128 K, 20 runs	20%	80%	−246.1	−246.1	55%	45%	−249.0	−248.8 (40%)
								−248.4 (50%)

a With the 16 K population, 1 run (4% of the outcomes) did not include either the 5 nt or the 3 nt stem structures.

We also modelled the co-transcriptional folding of other lengths of the 5′-leader sequence (not shown). Among these was the +1 to +173 length, to include the nucleolin binding site, the first known site for specific protein interaction in the 5′-leader. The sequence +1 to +700 was studied in order to encompass a region downstream of +414 known to affect leader cleavage [6] and including a U3 binding site [10]. The SNP at +139 did not significantly impact the folding of these sections of rRNA. Finally, we used another program, Mfold, to examine the gross structural characteristics of the large 5′-external transcribed spacer (+1 to +3,655) and observed strong conservation of the LL motif and no long-distance interactions with the nucleotides outside the LL sub-domain resulting from the presence or absence of the +139 SNP (not shown). Mfold results also showed potential for the formation of the same LL motif variants illustrated in Fig. 6 by the wild type and the +139 SNP sequences.

In addition we examined the nc-rRNA, which extends from about −1000 to at least +300.Its exact extent from the transcription start site downstream could not be determined because of overlap with the regular rRNA transcript. In all MPGAfold runs modelling the nc-rRNA from −1000 to +414, the LL motif was conserved and was not involved in interactions with sequences upstream of the transcription start site (not shown).

## Discussion

We report here that human epithelial cells, including lung cancer cells, like mouse fibroblasts, express a non-coding RNA from the intergenic region of the *rRNA* gene, and that a negative regulatory role is possibly implied, since there was a negative correlation between levels of the nc-rRNA and total rRNA in most of the human lung adenocarcinoma cell lines studied.

Further, we observed an interesting relationship between an nc-rRNA SNP and the nc-rRNA levels. The sequences of the multiple copies of *rRNA* genes are conserved among individuals of a species and within individuals, a phenomenon speculated to represent concerted evolution driven by multiple mechanisms, including intrachromosomal recombination and interchromosomal gene conversion [5], [11]. Some variations have been noted, especially in evolutionarily less conserved regions and in the 28S component of the gene [12], [13]. These changes mostly involved gain or loss of repetitive segments, although single nucleotide differences have also been noted [14]–[16]. No functional significance has yet been assigned to these differences in nuclear *rRNA*. However, several maternally-inherited point mutations in mitochondrial *rRNA* predispose children to ototoxicity of antibiotic aminoglycosides, by an incompletely understood mechanism involving mitochondrial membrane permeability and apoptosis [17]–[19].

In our study, the nc-rRNA was found to extend into the 5′- ETS leader region, past site +139, and differences in the frequency of +139 C among the cell lines correlated negatively with levels of nc-rRNA. On the basis of these observations, we postulate that the +139 C SNP has a negative regulatory effect on either the transcription rate of the nc-rRNA, or on its stability, and that the ncRNA in turn negatively regulates the rRNA.

In the mouse fibroblast cells studied by Mayer et al. [3], [4], the ncRNA expressed from the intergenic region of the *rRNA* gene caused a decrease in rRNA transcription as a result of interaction with the nucleolar remodelling complex and altered DNA and histone methylation. Specifically, the sequence −127 to −39 was implicated [4]. Sequences downstream of the transcription start site were not studied; whether these may operate by a comparable mechanism will require further study. Non-coding RNA has been implicated in control of gene expression by a variety of mechanisms [reviewed in 20]–[22]. In at least two instances, it has been reported that regulatory long non-coding RNA, initiated upstream, extends across the transcription initiation site, as observed in our results. These long nc-RNAs included an interfering transcript functioning as a negative repressor of the human dihydrofolate reductase gene [23], and a trans-promoter transcript activating the fructose-1,6-bisphosphatase gene in fission yeast [24].

There has been one publication pointing to a functional difference among SNPs in human *rRNA* [25]. In human HaCaT keratinocytes in culture, SNPs were localized in the region surrounding the transcription start site for *rRNA*. In chromatin immunoprecipitation assays, frequencies of SNPs were significantly different in *rRNA* gene regions immunoprecipitated with the Upstream Binding Factor (UBF) or with PolI, vs that combined with the transcription factor basonuclin. In the region −101 to −52, overall SNP frequency was quite low in the *rRNA* gene associated with UBF or PolI and significantly higher in the basonuclin-associated *rRNA*. This region encompasses the −96 SNP in our study, which occurred in a high percentage of the A549 clones. Lack of SNPs in the UBF-associated sequence is notable, in view of the promiscuity of binding of UBF throughout the *rRNA* gene and its regulatory regions [26]. It could be of interest to determine whether increased basonuclin/UBF binding ratio occurs in the A549 cell line. Basonuclin is expressed in epithelial as well as germ cells [27] and is highly increased in basal cell carcinoma of skin [28].

Further evidence for functional differences among *rRNA* gene variants comes from a study in mice [29]. Seven variants were identified based on restriction fragment length polymorphisms and included two SNPs in the promoter region. Variant SNPs in the 5′-ETS were utilized to examine expression in specific adult mouse tissues by quantitative PCR. Three variants were universally expressed (including one at different levels among tissues), two were not detected in any tissue, and two were found only in certain tissues. These results were consistent with earlier cytological assays of silver staining of nucleolar-organizing regions for different chromosomes in human cells: fibroblasts and leukocytes varied with regard to which chromosome clusters of *rRNA* genes were most active [30], and there were differences among clusters in response to the stimulating effect of serum [31].

The nc-rRNA, expressed from the intergenic region in mouse cells and interacting with the nucleolar remodelling complex, has recently been termed pRNA (promoter RNA), and was predicted by computer modelling to have a stable hairpin secondary structure, as was the comparable sequence from the human *rRNA* promoter [4]. When Mayer et al. [4] mutated tandem G-C pairs in the pRNA (at base pairs corresponding to sites −59 to −61 and −118 to −120 in the primary sequence used by us), they observed a major effect on secondary structure, elimination of a stem-loop configuration, and abrogation of specific binding to the TIP5 protein in the nucleolar remodelling complex. We checked whether a comparable alteration in secondary structure might accompany the +139 SNP. Computer modelling of the rRNA structures indicated that site +139 is localized in a stable, energetically favored stem structure we termed the LL motif. Co-transcriptional modelling indicated that the +139 U to C SNP had a relatively small but possibly significant effect on local structure, with the C substitution providing greater stability (overall lower free energy) and increased frequency of a 5-nt stem in place of a 3-nt stem. Whether this conformational difference might affect functional interactions of the LL motif will require further study.

Another possible explanation for the special effects of the site +139 SNP may be found in the DNA context: this site is located in a conserved core steroid response element, TGTTCT, for Class I hormone receptors, including those for glucocorticoids, androgens, mineralocorticoids, and progesterone. Change of T to C at site +139, the third position in this element, would likely eliminate hormone receptor binding: replacement of this T with A abolished response to all class I hormone receptors [32]. Also possibly relevant is the presence just downstream of +139, at +159 to +167, of a binding element for nucleolin, a major growth regulatory, RNA-binding protein [33], [34].

In addition to the +139 T/C SNP, we discovered 11 other sites where SNPs were found more frequently than expected by chance. None of these occurred often enough for correlations with cellular properties to be attempted. These SNPs might have functional significance, or may just be at sites that are particularly tolerant of altered sequence. Sequences in the region of the human *rRNA* gene which regulate transcription include the essential core promoter, approximately −45 to +18, which is essential for transcription, and an upstream control element starting at approximately −200, including the critical T_o_ element at −165 to −175 [35], [36]. No SNPs occurred in any of our lung cell lines from −13 to −4; changes in this region may not be functionally tolerated. We noted SNP hotspots in the upstream control element, at −181, −104, −96, and −72. Hotspot −104 is part of a binding site, TCGCGC, for the members of the E2F transcription factor family. Complex effects on *rRNA* transcription, both positive and negative, have been ascribed to E2F1, E2F4, and E2F6, when studied in transfected human lung adenocarcinoma H358 cells [37], [38]. It is possible that replacement of −104 T with C reduces repressive effects or enhances the induction effect of one or more E2F family members.

The polymorphisms at −96, all substituting a T for a C, eliminated a CpG site, where methylation could occur. The changes at −72, all substituting a C for a T, created a CpG site. In a recent study with human HeLa cancer cells, 27 CpG sites in the upstream control element and core promoter of *rRNA* were in general highly methylated, but in DNA immunoprecipitated with antibody to PolI these sites had reduced or absent methylation [39]. This included site −96 and one at −75. Possibly the loss of the −96 CpG could increase constitutive binding of PolI. This would be of particular importance in A549 cells, where nearly half of the clones showed this change. A comparative study of human liver cancers vs normal matching liver, found in the cancers, significant hypomethylation of all of the *rRNA* upstream CpG sites between −137 and −59, including −96 [40]. Also, in a DNase footprint assay, site −96 was located between two regions protected by UBF, and showed enhanced DNA cleavage [41].

It has also been noted that human and mouse external and internal transcribed spacers have a higher percentage of AA and GA dinucleotides, and a lower percentage of AT, CA, and TA dinucleotides, than expected by chance [8]. These may have special roles and might be expected to be conserved. Five of the hotspot SNPs in our study resulted in gain or loss of one of these five dinucleotide pairs. Of particular possible importance are the seven A/G SNPs at +52, which convert a GA and an AT to neutral GG and GT; and the nine G/A SNPs at +207, all in cell line H23, resulting in the creation of a GA dinucleotide.

In summary, an nc-rRNA, encompassing the *rRNA* gene transcription start site, was expressed in human lung adenocarcinoma cells and was negatively correlated with frequency of a T/C SNP at site +139 in the leader sequence of the 5′-ETS. For most cell lines, the nc-rRNA was also negatively correlated with the total 45S rRNA. Recently chemicals targeting transcription factors for *rRNA* have shown promise for treatment of cancer [42], [43]. Furthermore, possible reduced expression of rRNA in brain, due to hypermethylation, was linked to risk of suicide [44]. Most recently, it has been suggested that increased rRNA due to trisomy of chromosome 21 contributes to Down's syndrome [45]. Thus variability in rRNA has relevance for several human health issues. The functional significance of the SNP +139 requires further study. It had a local effect on the predicted distribution of the secondary structures, might alter binding of glucocorticoid receptors, and could interact with a nearby nucleolin binding site. There were also 11 additional SNP hotspots, some of which might be of functional importance. Specific contributions of *rRNA* gene variants deserve further study.

## Materials and Methods

### Human lung cell lines

HPL1D, a nontransformed immortalized cell line prepared from peripheral human lung epithelium [46], was obtained from Drs. T. Takahasi and A. Masuda. Human lung adenocarcinoma cell lines were obtained from the American Type Culture Collection (Manassas, VA) (A549, H23, H441, H727, H1355, H1395, H1703, H1792, H1944, H2023, H2030, H2122 and H2126). They were cultured in RPMI 1640 medium (Invitrogen, Carlsbad, CA) supplemented with 10% fetal calf serum (Gemini Bio-Products, Woodland, CA), 4 mM glutamine, and 100 units/mL penicillin and 100 ug/mL streptomycin (Invitrogen), at 37°C, 7% carbon dioxide. At confluence the cells' media was changed and then changed again the following day. Cells were harvested at 48 hrs post-confluence by treatment with 0.05% trypsin/0.02% EDTA (Invitrogen) and stored at −70° for later analysis. Post-confluent cells were chosen for study to maximize the comparability among the cell lines and to mimic conditions in a tumor mass in vivo.

### Preparation of DNA, PCR, cloning and dideoxy sequencing

DNA was extracted using Easy-DNA kits (Invitrogen). A 50 ng DNA aliquot was used for amplification with a PlatinumTaq PCR kit (Invitrogen). The −408 to +326 region of human *45S rRNA* (GenBank Accession AL592188) was amplified by PCR, using forward primer: 5′-gcgatcctttctggcgagtcc-3′ (−408 to −389) and reverse primer: 5′-gacgagaacgcctgacacgca-3′ (+307 to +326). In brief, amplification was carried out in a final 50 µl PCR mixture containing 20 mM Tris-HCL (pH 8.0), 50 mM KCl, 1.5 mM MgCl_2_, 0.2 mM deoxynucleotide triphosphates, 0.2 µM primers, 10% dimethyl sulfoxide, and 1.25 U PlatinumTaq polymerase. PCR was performed with an initial denaturation at 94°C for 2 min followed by 40 cycles of denaturation (94°C for 30 s), annealing (67°C, 30 s), and extension (72°C, 1 min). Amplified products were resolved by electrophoresis using 10% polyacrylamide gels (Invitrogen) and specific amplification verified after ethidium bromide (0.5 µg/ml) fluorescence staining and imaging analysis (UVP Inc., Upland, CA).

Cloning was performed using a TOPO Cloning kit (Invitrogen) to obtain individual sequence clones for Sanger's dideoxy DNA sequencing. In brief, approximately 1–2 µl of fresh PCR product were used according to the manufacturer's instructions. The bacteria carrying plasmids of correct PCR product inserts were positively selected in culture plates in the presence of ampicillin and kanamycin antibiotics for 24 hrs. For each PCR sample, colonies were randomly picked, lysed, PCR amplified using M13 vector primers provided in the cloning kit, purified with GFX DNA binding spin-columns (Amersham Pharmacia Biotech Inc., Piscataway, NJ), and sequenced by fluorescence-based dideoxy sequencing with an ABI 373 Sequencer (Applied Biosystems, Foster City, CA). SNPs were identified by a SNP Report program, which was written in Paradox version 4 (Borland, Austin, TX). It converts output from the GenBank “Blast 2 Sequences” online alignment software (www.ncbi.nlm.nih.gov) to a manageable database of identified sequence variants. The program also allows users to generate various SNP reports, including type of base change, its location, and frequency pertinent to categories (such as cell line and cancer vs. non-cancer) specified by users. Blast2 alignment results from PCR clones compared with the wild-type reference sequence were first saved as text files. Each text file was given a specific clone number, cell line name, and date. Gap(s) in the alignment, indicative of base change(s), were recognized and subsequently assigned according to nucleotide type and location by the SNP Report program.

### RNA analysis

Total RNA was isolated with the Versagene RNA Purification System (Gentra Systems, Minneapolis, MN) or RNeasy Mini kit (Qiagen, Germantown, MD) according to the manufacturers' protocols. DNase treatment was performed to remove residual DNA from the RNA samples by use of the recombinant DNase I as specified in the protocol supplied with the Ambion DNA-free Kit (Applied Biosystems, Foster City, CA). The treated samples were washed twice with diethyl pyrocarbonate (DEPC)-treated water at 14,000×g in Microcon 50 spin columns (Millipore, Bedford MA) and finally eluted in 40–50 uL of DEPC-treated water. Total RNA was quantified spectrophotometrically at 260 nm and diluted to a working concentration of 50 ng/uL.

Primers for reverse transcription (RT) and for PCR were selected using Oligo software, version 6 (Molecular Biology Insights, Cascade, CO) and PSQ Assay Design (Biotage, Uppsala, Sweden). RT was carried out using SuperScript III (Invitrogen) and gene-specific primers to generate cDNAs according to the manufacturer's protocol. The primer used for RT determination of a 5′ rRNA external transcribed spacer region, indicative of rRNA level, was cgtctcgtctcgtctcac. Sequences assessed for nc-rRNA are shown in Figure 2. Primers used for RT of these regions were: Region A, aataacccggcggcccaaaa; Region B, ccctctggtcaaccccaggacacac; Region C, gcccgtgtctccagagcg; Region D, ccacggggactcggaaacgaatt; Region E, cagcctctcccccacataaacctgc; Region F, gcgaaagcgaaaccgtgagtcga; Region G, agacacacggagaggcagaatgcg; Region H, agaaggaaacacagggaacgaaagagaaat; and Region I, gggtgggtcagagacccgga. PCR primer pairs were: rRNA transcript, gcccgtcgtgctgccct/gggcgggaacgacacacca; Region A, ccccctgcgtgtggcacg/aataacccggcggccaaaa; Region B, cggccttcggtccctcgtgtg/ccctctggtcaaccccaggacacac; Region C, gcgatcctttctggcgagtc/gcccgtgtctccagagcg; Region D, tgtgggggagaggctgtcgct/ccacggggactcggaaacgaatt; Region E, ggcctcgggaagagcttctcgac/cagcctctcccccacataaacctgc; Region F, gcattctgcctctccgtgtgtctgc/gcgaaagcgaaaccgtgagtcga; Region G, ctctctttctgtccttttttccttcgtgc/agacacacggagaggcagaatgcg; Region H, acatcgctctataaagaagggatcgtcga/agaaggaaacacagggaacgaaagagaaat; and Region I, ccccctgcgtgtggcacg/gggtgggtcagagacccgga for ncRNA.

Quantitative real-time polymerase reactions (qPCR) for rRNA and for nc-rRNA region A were performed with the Chromo4 System (MJ Research, Waltham, MA). The qPCR 20 µL mixture contained 0.5 µM gene-specific upper and lower primers, 10 µl of 2X SYBR Green PCR Mix (Qiagen), and a 2 µL aliquot of cDNA. Amplification was carried out at 95°C for 15 minutes followed by cycling at 94°C for 15 seconds, 65°C for 20 seconds, and 72°C for 30 seconds. The Ct values were obtained using the Opticon Monitor 2 software (MJ Research). Amounts of PCR products were estimated from the fluorescence intensity as 2^−Ct^. For determination of nc-rRNA upstream regions, the same protocol was used for regions D and I as above. Two-step PCR was performed with denaturation at 94°C for 15 seconds and annealing/extension at 68°C for 90 seconds for the 7 remaining regions. For regions F, G and H, additional two-step PCR reactions were carried out with annealing/extension at 72°C for 45 seconds to exclude interference of amplification due to GC-rich secondary structure.

It is customary to normalize RNA expression for genes of interest to that of a housekeeping gene of non-variable expression, and in fact rRNA is often used for this purpose. We tested a number of genes, *GAPDH*, *PSMB2*, *RPL32*, and *PPIB*, evaluated previously as internal controls for lung cells [47], but none of these were useful due to large variations between cell lines and/or between experiments (data not shown). Instead, we minimized the potential for error due to loading and RT efficiency variations by quantifying total RNA based on 2 or more repeated spectrophotometer measurements and by performing multiple (minimum of 3) RT-PCR runs. Reproducible data were obtained from these repeated experiments.

The presence and relative amounts of SNP alleles in rRNA were determined by pyrosequencing. rRNA was converted to cDNA as described above, except that the total PCR cycle number was limited to 30 to avoid product saturation, and different primers were used, 5′-biotinylated gacctgtcgctgggagaggttg and regular agtggggacgctcggacg. To prepare the template for pyrosequencing, biotinylated PCR products in a 10 µL aliquot were captured in 2 µL streptavidin-sepharose beads (Amersham Biosciences, Pisctaway, NJ) mixed with 40 µL binding buffer (10 mM Tris-HCl, 2 M NaCl, 1 mM EDTA, and 0.1% Tween-20, pH 7.6) and 35 µL doubly distilled water. After denaturation in 0.2 M NaOH and wash in 10 mM Tris-acetate, pH 7.6, the single-stranded template was annealed with 3 pmol sequencing primer (tcggacgcacgagaga) in a buffer containing 20 mM Tris-acetate, pH 7.6, with 2 mM magnesium diacetate. The rRNA transcript variant quantification was performed in PSQ 96 HS System (Biotage) using PyroMark MD software and PyroGold reagents.

### Statistical methods

Data in this study were analyzed with both parametric and nonparametric methods. The nonparametric runs test was used to determine whether there were stretches of sequences in which the frequencies of SNPs were higher (or lower) than predicted by chance. Contingency table analyses were used to compare selective SNPs between cancer and HPL1D non-transformed lines. Several hypotheses were formulated to investigate the randomness of SNP counts. Pearson chi-square tests were used to test for homogeneity of counts across cell lines. To investigate whether SNPs were more frequent in certain specific intervals or strata of sites than in the complimentary strata, Poisson regression analyses with offset parameters equal to the number of sites were performed so that the SNP rates could be compared in the suspected hot spot intervals to the rates in the complimentary intervals. To investigate whether SNPs were more likely to occur at individual sites, tail probabilities from the Poisson distribution were calculated to determine if the number of occurrences were larger than expected by chance. For analyses involving Poisson regression, we routinely evaluated the assumptions underlying model fits and included over-dispersion parameters when appropriate. With contingency table analyses, asymptotic and exact methods were employed. In all cases, probabilities extracted from exact methods were in agreement with asymptotic results. For simplicity, asymptotic results are reported. Comparisons of total rRNA and nc-rRNA to HPL1D were conducted with independent t tests. Linear regression and rank correlation analysis were utilized to determine the relationship between nc-rRNA and total rRNA. All statistical tests were two-sided. Probability values (*P*) less than 0.05 were considered significant.

### RNA secondary structure prediction

To predict the secondary structure of fragments of rRNA, we used our massively parallel genetic algorithm, MPGAfold, involving populations of thousands of structures [48]–[51]. MPGAfold is a stochastic algorithm, which tends to stress the most significant RNA conformations among its solutions, including biologically significant transient or intermediate structures [50], [52]–[55]. In brief, using the free energy of RNA secondary structures for a given input sequence as the fitness function, the algorithm evolves a population of RNA conformers by exchanging parts of the conformers, inserting mutations in structural elements and selecting the best fit individual conformations resulting from these operations. The fitness of the dominant conformations increases with size of the population, with the significant suboptimal (transitional) conformations being best captured by the lower population level runs. For the present analyses, results of folding runs at populations of 16 K, 64 K, and 128 K were investigated. We have also employed the ability of the algorithm to simulate co-transcriptional folding, i.e. folding of an elongating sequence. In this mode the folding structures evolve in a changing sequence context and local and transitional motifs are more likely to form and persist longer. Based on our experience with the algorithm and the experimental data supporting its results in other studies [50], [52]–[55] we used the elongation rate of two nucleotides per MPGAfold generation. The analysis of the folding predictions was performed with StructureLab [56]–[58]. The long sequence +1 to +3,655, corresponding to the entire rRNA 5′-external transcribed spacer, was modelled with Mfold [59], [60]. The reference sequence, GenBank accession entry AL592188, was used for these operations.

## Supporting Information

Table S1 (0.87 MB DOC)
