# Supplementary material for: An Intergenic Non-Coding rRNA Correlated with Expression of the rRNA and Frequency of an rRNA Single Nucleotide Polymorphism in Lung Cancer Cells
Source: PLoS One. 2009 Oct 19;4(10):e7505. doi: 10.1371/journal.pone.0007505 (PMC2759515; doi:10.1371/journal.pone.0007505)
Supplement: Table S1 — (0.87 MB DOC) [file pone.0007505.s001.doc]

Table S1. *rRNA* SNPs flanking the transcription start site in 7 lung cell lines.

|  | Cell line |  | A549 | H441 | H23 | H1792 | H2030 | H2122 | HPL |
| --- | --- | --- | --- | --- | --- | --- | --- | --- | --- |
|  | Total clones |  | 54 | 36 | 48 | 52 | 56 | 65 | 51 |
|  | SNP+ clones |  | 47 | 29 | 43 | 41 | 40 | 44 | 38 |
| Nucleotide^a^ | Site^b^ |  |  |  |  |  |  |  |  |
| 149008C | -388 |  |  |  |  |  |  |  |  |
| 149009C | -387 |  |  |  |  |  |  | 2del |  |
| 149010G | -386 |  |  |  |  |  |  |  |  |
| 149011T | -385 |  |  | A |  | C |  | C |  |
| 149012G | -384 |  |  |  |  |  |  |  |  |
| 149013C | -383 |  |  | G |  |  |  |  |  |
| 149014G | -382 |  |  |  |  |  |  |  |  |
| 149015G | -381 |  |  |  |  | A |  |  |  |
| 149016A | -380 |  |  |  | C |  |  |  |  |
| 149017G | -379 |  |  |  |  |  |  |  |  |
| 149018T | -378 |  |  |  |  |  |  | C |  |
| 149019C | -377 |  |  |  |  |  |  |  |  |
| 149020G | -376 |  |  |  |  |  |  |  |  |
| 149021G | -375 |  |  |  |  |  |  |  |  |
| 149022A | -374 |  | T |  |  |  |  | G |  |
| 149023G | -373 |  |  |  |  |  |  | A |  |
| 149024A | -372 |  | G | G |  |  |  |  |  |
| 149025G | -371 |  |  |  |  |  |  |  |  |
| 149026C | -370 |  |  |  |  |  |  |  |  |
| 149027G | -369 |  |  |  |  |  |  |  |  |
| 149028C | -368 |  |  |  |  |  |  |  |  |
| 149029T | -367 |  |  |  |  | C |  | C |  |
| 149030C | -366 |  | ins T | ins T |  |  |  |  |  |
| 149031C | -365 |  |  |  |  |  |  |  |  |
| 149032C | -364 |  |  |  |  |  |  |  |  |
| 149033T | -363 |  |  |  |  |  |  |  |  |
| 149034G | -362 |  |  |  |  |  |  | A |  |
| 149035A | -361 |  |  |  |  |  |  |  | T |
| 149036G | -360 |  |  |  |  |  |  |  |  |
| 149037C | -359 |  |  | T |  |  |  |  |  |
| 149038G | -358 |  |  |  |  |  |  |  |  |
| 149039C | -357 |  |  |  |  |  | T |  |  |
| 149040G | -356 |  |  |  |  |  |  |  |  |
| 149041C | -355 |  |  |  |  |  |  |  |  |
| 149042G | -354 |  |  |  |  |  |  |  |  |
| 149043T | -353 |  |  |  |  |  | A |  | C |
| 149044G | -352 |  |  |  |  |  |  |  |  |
| 149045C | -351 |  |  |  |  |  |  |  |  |
| 149046G | -350 |  |  |  |  |  |  |  |  |
| 149047G | -349 |  |  |  |  |  |  |  |  |
| 149048C | -348 |  |  |  |  |  |  |  |  |
| 149049C | -347 |  |  |  |  |  |  |  |  |
| 149050C | -346 |  |  |  |  |  |  |  |  |
| 149051G | -345 |  |  |  |  |  |  |  |  |
| 149052A | -344 |  |  |  |  |  |  |  |  |
| 149053G | -343 |  |  |  |  |  |  |  |  |
| 149054A | -342 |  | T | G |  |  |  | G, T | T |
| 149055G | -341 |  |  |  |  |  |  |  |  |
| 149056G | -340 |  |  |  |  |  |  |  |  |
| 149057T | -339 |  |  |  |  |  |  |  | C |
| 149058C | -338 |  |  |  |  |  |  |  |  |
| 149059G | -337 |  |  |  |  |  |  |  |  |
| 149060C | -336 |  |  |  |  |  |  |  |  |
| 149061G | -335 |  |  |  |  |  |  |  |  |
| 149062C | -334 |  |  |  |  |  |  |  |  |
| 149063C | -333 |  | T |  | T |  |  |  | T |
| 149064T | -332 |  | C | A |  |  | C |  |  |
| 149065G | -331 |  |  |  |  |  | ins T |  |  |
| 149066G | -330 |  |  |  |  |  |  |  |  |
| 149067C | -329 |  |  |  |  |  |  |  | G |
| 149068C | -328 |  |  | G |  |  |  |  | G |
| 149069G | -327 |  |  |  |  |  |  |  |  |
| 149070G | -326 |  |  |  |  |  |  |  |  |
| 149071C | -325 |  |  |  |  | T |  |  |  |
| 149072C | -324 |  |  |  |  | T |  |  |  |
| 149073T | -323 |  |  |  | C |  |  | C |  |
| 149074T | -322 |  |  |  |  | C |  |  |  |
| 149075C | -321 |  |  | ins T |  |  |  | ins T |  |
| 149076G | -320 |  |  | T |  |  |  |  |  |
| 149077G | -319 |  |  |  |  |  |  |  |  |
| 149078T | -318 |  | A |  |  | C |  | C |  |
| 149079C | -317 |  |  |  |  |  |  |  |  |
| 149080C | -316 |  |  |  |  | 2-A |  |  |  |
| 149081C | -315 |  |  |  |  |  |  |  |  |
| 149082T | -314 |  |  |  |  |  |  | C |  |
| 149083C | -313 |  |  |  |  |  |  |  |  |
| 149084G | -312 |  |  |  |  |  |  |  |  |
| 149085T | -311 |  |  | C | 2 C | C |  |  |  |
| 149086G | -310 |  |  |  |  |  |  |  |  |
| 149087T | -309 |  |  | C |  |  |  | C |  |
| 149088G | -308 |  |  | A |  |  |  |  |  |
| 149089T | -307 |  |  |  |  |  |  |  |  |
| 149090C | -306 |  |  |  |  |  |  |  |  |
| 149091C | -305 |  |  | T |  |  |  |  |  |
| 149092C | -304 |  |  |  |  |  |  |  | A |
| 149093G | -303 |  |  |  |  |  |  |  |  |
| 149094G | -302 |  |  |  |  |  | A |  |  |
| 149095T | -301 |  |  |  |  | C |  | A |  |
| 149096C | -300 |  |  |  |  |  |  |  |  |
| 149097G | -299 |  |  |  |  |  |  | A |  |
| 149098T | -298 |  |  |  |  |  |  |  |  |
| 149099A | -297 |  |  |  | G |  |  |  |  |
| 149100G | -296 |  |  |  | A |  |  |  |  |
| 149101G | -295 |  |  |  |  |  |  |  |  |
| 149102A | -294 |  |  |  | C |  |  |  |  |
| 149103G | -293 |  |  |  |  |  | A |  |  |
| 149104G | -292 |  |  |  |  |  |  |  |  |
| 149105G | -291 |  |  |  |  |  |  |  |  |
| 149106G | -290 |  |  |  |  |  |  |  |  |
| 149107C | -289 |  |  |  |  |  |  |  |  |
| 149108C | -288 |  |  | G | T |  |  |  |  |
| 149109G | -287 |  |  |  |  |  |  |  |  |
| 149110G | -286 |  |  | C |  |  |  |  |  |
| 149111C | -285 |  |  |  |  |  |  |  | G,T |
| 149112C | -284 |  | T |  |  |  |  |  |  |
| 149113G | -283 |  |  |  |  |  |  |  |  |
| 149114A | -282 |  |  |  | G |  |  |  |  |
| 149115A | -281 |  |  |  |  |  |  |  |  |
| 149116A | -280 |  |  | G |  |  |  |  |  |
| 149117A | -279 |  |  | T | G,G |  | G | del |  |
| 149118T | -278 |  |  | G |  |  | C | C |  |
| 149119G | -277 |  |  | C |  |  |  |  |  |
| 149120C | -276 |  |  | T |  |  |  |  |  |
| 149121T | -275 |  | C |  |  |  |  |  |  |
| 149122T | -274 |  |  |  | A,C |  | A, C |  |  |
| 149123C | -273 |  |  |  |  |  |  |  |  |
| 149124C | -272 |  |  |  |  |  |  |  |  |
| 149125G | -271 |  |  |  |  |  |  |  | A |
| 149126G | -270 |  |  |  |  |  |  |  |  |
| 149127C | -269 |  |  |  |  |  |  |  |  |
| 149128T | -268 |  |  |  | C |  |  | C |  |
| 149129C | -267 |  |  |  |  |  |  |  |  |
| 149130C | -266 |  |  |  |  |  |  |  |  |
| 149131C | -265 |  |  |  |  |  |  |  |  |
| 149132G | -264 |  |  |  |  |  |  |  |  |
| 149133C | -263 |  |  |  |  |  |  | T |  |
| 149134T | -262 |  |  |  |  |  |  |  |  |
| 149135C | -261 |  |  |  |  |  |  |  |  |
| 149136T | -260 |  |  |  |  |  |  |  | G |
| 149137G | -259 |  |  |  |  |  |  |  |  |
| 149138G | -258 |  |  |  |  |  |  |  |  |
| 149139A | -257 |  |  |  |  |  | T |  |  |
| 149140G | -256 |  |  |  |  |  |  |  |  |
| 149141A | -255 |  |  |  |  |  |  | T |  |
| 149142C | -254 |  |  |  |  |  |  |  |  |
| 149143A | -253 |  |  |  |  | G | T |  |  |
| 149144C | -252 |  |  |  |  |  |  |  |  |
| 149145G | -251 |  |  |  |  |  |  |  |  |
| 149146G | -250 |  |  |  |  |  |  |  |  |
| 149147G | -249 |  | 2 C |  |  |  |  |  |  |
| 149148C | -248 |  |  |  |  |  |  |  |  |
| 149149C | -247 |  |  |  |  |  |  |  |  |
| 149150G | -246 |  |  |  | ins C |  |  | A |  |
| 149151G | -245 |  |  |  |  |  |  |  |  |
| 149152C | -244 |  |  |  | G |  |  |  |  |
| 149153C | -243 |  |  |  |  |  |  |  |  |
| 149154C | -242 |  |  |  |  |  |  |  |  |
| 149155C | -241 |  |  |  |  |  |  |  |  |
| 149156C | -240 |  | T |  |  |  |  |  | T |
| 149157T | -239 |  |  | ins C | 2 ins C | ins C |  | C |  |
| 149158G | -238 |  |  | T | ins T | T |  |  |  |
| 149159C | -237 |  |  |  |  | G |  |  |  |
| 149160G | -236 |  |  |  | C | C |  |  | A |
| 149161T | -235 |  |  |  |  | G |  |  |  |
| 149162G | -234 |  | del | del | 2 del | T |  |  | 4 del |
| 149163T | -233 |  | A, del | del | A, 2 del | del | C | del | del |
| 149164G | -232 |  |  |  | T |  |  |  |  |
| 149165G | -231 |  |  |  |  | A |  |  |  |
| 149166C | -230 |  |  |  | A, G |  |  |  |  |
| 149167A | -229 |  |  |  | G,C |  |  |  | G, T |
| 149168C | -228 |  |  |  | A |  |  |  | 2 T |
| 149169G | -227 |  |  |  |  |  |  |  |  |
| 149170G | -226 |  |  |  |  |  |  |  |  |
| 149171G | -225 |  |  |  |  |  |  |  |  |
| 149172C | -224 |  |  |  |  |  |  |  |  |
| 149173G | -223 |  |  |  |  |  |  |  |  |
| 149174G | -222 |  |  |  |  | C |  |  |  |
| 149175C | -221 |  |  |  |  |  |  |  |  |
| 149176C | -220 |  |  |  |  |  |  |  |  |
| 149177G | -219 |  |  |  |  |  |  |  |  |
| 149178G | -218 |  |  |  |  |  |  |  |  |
| 149179G | -217 |  |  |  | A |  |  |  |  |
| 149180A | -216 |  |  |  |  | 2 G |  | G |  |
| 149181G | -215 |  |  |  | T |  |  |  | T |
| 149182G | -214 |  |  |  |  |  |  |  |  |
| 149183G | -213 |  |  |  |  |  |  |  |  |
| 149184C | -212 |  | T |  |  |  |  |  |  |
| 149185G | -211 |  |  |  |  |  |  |  |  |
| 149186T | -210 |  |  |  |  |  |  |  |  |
| 149187C | -209 |  |  |  |  |  |  |  |  |
| 149188C | -208 |  |  |  |  |  |  |  |  |
| 149189C | -207 |  |  |  |  |  |  |  |  |
| 149190C | -206 |  |  |  |  | 3 T | T |  |  |
| 149191G | -205 |  |  |  |  | A |  |  |  |
| 149192G | -204 |  |  |  |  |  |  |  |  |
| 149193C | -203 |  |  |  |  |  |  |  |  |
| 149194C | -202 |  |  |  |  |  |  |  |  |
| 149195C | -201 |  | A |  |  |  |  |  |  |
| 149196G | -200 |  |  |  |  |  |  |  |  |
| 149197G | -199 |  |  |  |  | A |  |  |  |
| 149198C | -198 |  |  |  |  |  |  |  |  |
| 149199G | -197 |  |  |  |  | A |  |  |  |
| 149200C | -196 |  |  |  |  |  |  |  |  |
| 149201T | -195 |  |  |  | C |  |  |  |  |
| 149202G | -194 |  |  |  |  |  |  |  |  |
| 149203C | -193 |  |  |  |  |  |  |  |  |
| 149204T | -192 |  |  |  | C |  |  |  |  |
| 149205C | -191 |  |  |  |  |  |  |  |  |
| 149206C | -190 |  |  |  |  |  |  |  |  |
| 149207C | -189 |  |  |  | A |  |  |  |  |
| 149208G | -188 |  |  |  |  |  |  |  |  |
| 149209C | -187 |  |  |  |  |  |  |  |  |
| 149210G | -186 |  |  |  |  |  |  |  | A |
| 149211T | -185 |  |  |  | C |  |  |  |  |
| 149212G | -184 |  |  |  |  |  |  |  |  |
| 149213T | -183 |  | C |  |  | 2 C |  |  |  |
| 149214G | -182 |  |  |  |  |  | 3 del |  |  |
| 149215T | -181 |  |  |  |  |  | 3 del |  | A, 2C |
| 149216C | -180 |  |  |  |  |  |  |  |  |
| 149217C | -179 |  |  | T |  |  |  |  |  |
| 149218T | -178 |  |  |  | C |  | C |  |  |
| 149219G | -177 |  |  |  |  |  |  |  |  |
| 149220G | -176 |  |  |  |  |  |  |  |  |
| 149221G | -175 |  |  |  |  |  |  |  |  |
| 149222G | -174 |  |  |  | A |  |  |  | ins G |
| 149223T | -173 |  |  |  |  |  | C |  | G |
| 149224T | -172 |  |  |  |  |  | A | A,C |  |
| 149225G | -171 |  |  |  |  |  |  |  |  |
| 149226A | -170 |  |  |  |  |  |  |  |  |
| 149227C | -169 |  |  |  |  |  |  |  |  |
| 149228C | -168 |  |  |  |  |  |  |  |  |
| 149229A | -167 |  |  |  |  | 2 G |  | 2 T |  |
| 149230G | -166 |  |  |  |  |  |  |  |  |
| 149231A | -165 |  | G |  |  | 2 G | G |  |  |
| 149232G | -164 |  |  |  |  |  |  |  |  |
| 149233G | -163 |  |  |  |  |  |  |  |  |
| 149234G | -162 |  |  |  |  |  |  |  |  |
| 149235C | -161 |  |  |  |  |  |  |  |  |
| 149236C | -160 |  |  |  |  | G |  |  |  |
| 149237C | -159 |  |  |  |  |  | A |  |  |
| 149238C | -158 |  |  |  |  |  |  |  |  |
| 149239G | -157 |  | C |  |  |  |  |  |  |
| 149240G | -156 |  |  |  |  |  |  |  |  |
| 149241G | -155 |  |  |  |  |  |  |  |  |
| 149242C | -154 |  |  |  |  |  |  |  |  |
| 149243G | -153 |  |  |  |  |  |  |  |  |
| 149244C | -152 |  |  |  |  |  |  |  |  |
| 149245T | -151 |  | del |  |  | C |  |  |  |
| 149246C | -150 |  | T, del |  |  |  |  |  |  |
| 149247C | -149 |  |  |  |  | A, T |  |  |  |
| 149248G | -148 |  |  |  |  |  |  |  | A |
| 149249T | -147 |  |  |  |  |  |  |  |  |
| 149250G | -146 |  |  |  |  |  |  |  |  |
| 149251T | -145 |  |  |  | C |  |  |  |  |
| 149252G | -144 |  |  | A |  |  | del |  |  |
| 149253T | -143 |  | A, C | del |  |  | del | A |  |
| 149254G | -142 |  |  | del |  |  |  |  |  |
| 149255G | -141 |  | A |  |  |  |  | 2 C |  |
| 149256C | -140 |  |  |  |  |  |  |  |  |
| 149257T | -139 |  |  |  | C | 2 C | A |  |  |
| 149258G | -138 |  |  |  |  |  |  |  |  |
| 149259C | -137 |  |  |  |  |  |  |  |  |
| 149260G | -136 |  |  | A | A |  |  |  | A |
| 149261A | -135 |  |  |  |  |  |  |  |  |
| 149262T | -134 |  |  |  |  |  |  |  |  |
| 149263G | -133 |  |  |  |  |  |  |  |  |
| 149264G | -132 |  |  |  |  | A |  |  |  |
| 149265T | -131 |  | C |  |  | C |  |  |  |
| 149266G | -130 |  |  |  |  |  |  |  |  |
| 149267G | -129 |  | del |  |  |  |  |  |  |
| 149268C | -128 |  |  |  |  |  |  |  |  |
| 149269G | -127 |  |  |  | A |  |  |  |  |
| 149270T | -126 |  |  |  |  |  | C |  |  |
| 149271T | -125 |  |  |  |  |  |  | C | A |
| 149272T | -124 |  |  |  | C |  |  |  | C |
| 149273T | -123 |  |  |  |  |  | C | G |  |
| 149274T | -122 |  |  |  | A, C |  | C |  | del |
| 149275G | -121 |  |  |  |  | T |  |  |  |
| 149276G | -120 |  |  |  |  |  |  |  |  |
| 149277G | -119 |  |  |  |  |  |  |  |  |
| 149278G | -118 |  |  |  | C |  |  | C |  |
| 149279A | -117 |  | G |  |  | G | T | ins G |  |
| 149280C | -116 |  |  |  |  |  |  |  |  |
| 149281A | -115 |  |  |  | G |  |  |  |  |
| 149282G | -114 |  |  |  | A |  | A |  |  |
| 149283G | -113 |  |  |  |  | A |  |  |  |
| 149284T | -112 |  |  | A |  |  | C |  |  |
| 149285G | -111 |  |  |  |  | A |  |  |  |
| 149286T | -110 |  |  |  |  | G | 2 C |  |  |
| 149287C | -109 |  |  |  |  | A |  |  |  |
| 149288C | -108 |  |  |  |  |  |  |  |  |
| 149289G | -107 |  |  |  |  |  |  | 2-A |  |
| 149290T | -106 |  |  | A |  |  |  |  |  |
| 149291G | -105 |  |  |  |  |  |  |  |  |
| 149292T | -104 |  | 5C |  |  |  |  | A |  |
| 149293C | -103 |  |  |  |  |  |  |  |  |
| 149294G | -102 |  |  |  |  |  | C |  |  |
| 149295C | -101 |  |  |  |  |  |  |  |  |
| 149296G | -100 |  |  |  |  |  |  |  |  |
| 149297C | -99 |  |  |  |  |  |  |  |  |
| 149298G | -98 |  |  |  |  |  |  |  |  |
| 149299T | -97 |  |  |  |  |  |  |  |  |
| 149300C | -96 |  | 22T |  |  | 2 T | 4 T |  |  |
| 149301G | -95 |  |  | A |  |  |  | A |  |
| 149302C | -94 |  |  |  |  |  |  |  |  |
| 149303C | -93 |  |  |  |  |  |  |  |  |
| 149304T | -92 |  |  |  |  | A |  |  | C |
| 149305G | -91 |  |  |  |  |  |  |  |  |
| 149306G | -90 |  |  |  |  |  |  |  |  |
| 149307G | -89 |  |  |  |  |  | C |  |  |
| 149308C | -88 |  |  |  |  |  |  |  |  |
| 149309C | -87 |  | A |  |  |  |  | A |  |
| 149310G | -86 |  |  |  |  |  |  |  |  |
| 149311G | -85 |  |  |  |  |  |  |  |  |
| 149312C | -84 |  |  |  |  |  | T |  |  |
| 149313G | -83 |  |  |  |  |  |  |  |  |
| 149314G | -82 |  |  |  |  |  |  |  |  |
| 149315C | -81 |  |  |  |  |  |  |  |  |
| 149316G | -80 |  |  |  |  |  |  |  |  |
| 149317T | -79 |  | A |  |  |  |  |  |  |
| 149318G | -78 |  |  |  |  |  |  |  |  |
| 149319G | -77 |  |  |  |  |  |  |  |  |
| 149320T | -76 |  |  |  |  | 2 C |  |  |  |
| 149321C | -75 |  |  |  |  |  |  | T | A |
| 149322G | -74 |  |  |  |  |  |  |  |  |
| 149323G | -73 |  |  |  |  |  | 3 T |  |  |
| 149324T | -72 |  |  |  |  | C | 2 C | 3 C |  |
| 149325G | -71 |  |  |  |  | T |  |  |  |
| 149326A | -70 |  |  |  |  | G |  | G |  |
| 149327C | -69 |  |  |  |  | T |  |  |  |
| 149328G | -68 |  |  |  |  |  |  |  |  |
| 149329C | -67 |  |  |  |  |  |  |  |  |
| 149330G | -66 |  |  |  |  |  | A | 2-A |  |
| 149331A | -65 |  |  |  | G, T |  |  |  |  |
| 149332C | -64 |  |  |  |  |  |  |  |  |
| 149333C | -63 |  |  |  |  | T |  |  |  |
| 149334T | -62 |  | 2 C |  |  |  |  |  |  |
| 149335C | -61 |  |  |  |  | T |  |  |  |
| 149336C | -60 |  |  |  |  |  |  |  |  |
| 149337C | -59 |  |  |  | T |  | T |  |  |
| 149338G | -58 |  | A |  |  |  |  | ins C |  |
| 149339G | -57 |  |  |  |  |  |  |  |  |
| 149340C | -56 |  |  |  |  |  |  | G |  |
| 149341C | -55 |  |  |  |  | A |  |  |  |
| 149342C | -54 |  |  |  |  |  |  | 3 G |  |
| 149343C | -53 |  |  |  |  |  |  | ins G |  |
| 149344G | -52 |  | ins C |  |  |  | ins C |  |  |
| 149345G | -51 |  |  |  |  |  |  |  |  |
| 149346G | -50 |  |  |  |  |  |  |  |  |
| 149347G | -49 |  |  |  |  |  |  |  |  |
| 149348G | -48 |  |  |  |  |  |  |  |  |
| 149349A | -47 |  |  |  |  | G |  | T | 2 G |
| 149350G | -46 |  |  |  |  |  |  |  |  |
| 149351G | -45 |  |  |  |  |  |  |  |  |
| 149352T | -44 |  |  |  |  |  |  |  |  |
| 149353A | -43 |  | C |  |  |  |  |  |  |
| 149354T | -42 |  |  |  |  |  |  |  |  |
| 149355A | -41 |  |  |  |  |  |  |  |  |
| 149356T | -40 |  |  |  |  | 2 C |  |  |  |
| 149357C | -39 |  |  |  |  |  |  |  |  |
| 149358T | -38 |  | C |  |  |  |  |  |  |
| 149359T | -37 |  | C |  |  |  |  |  |  |
| 149360T | -36 |  |  |  |  | C |  | C |  |
| 149361C | -35 |  |  |  |  |  |  |  |  |
| 149362G | -34 |  |  |  |  |  |  |  |  |
| 149363C | -33 |  |  |  |  |  |  |  |  |
| 149364T | -32 |  |  |  |  |  |  |  | C |
| 149365C | -31 |  |  |  |  |  |  |  |  |
| 149366C | -30 |  |  |  |  |  |  |  |  |
| 149367G | -29 |  |  |  |  |  |  |  |  |
| 149368A | -28 |  |  |  | G |  |  | T | G |
| 149369G | -27 |  |  |  |  |  |  |  |  |
| 149370T | -26 |  |  |  |  |  |  |  |  |
| 149371C | -25 |  |  | T | T |  |  | A |  |
| 149372G | -24 |  | A |  | A |  |  |  |  |
| 149373G | -23 |  |  |  | A |  |  |  |  |
| 149374C | -22 |  |  |  |  |  |  |  |  |
| 149375A | -21 |  |  |  |  |  |  |  |  |
| 149376T | -20 |  |  |  |  |  | C | C |  |
| 149377T | -19 |  | C |  | C |  |  | C |  |
| 149378T | -18 |  |  |  |  |  |  |  |  |
| 149379T | -17 |  | C |  |  |  |  | 3 C |  |
| 149380G | -16 |  |  |  | A |  |  |  | T |
| 149381G | -15 |  |  |  |  |  | C |  |  |
| 149382G | -14 |  | del | T |  |  |  |  |  |
| 149383C | -13 |  |  |  |  |  |  |  |  |
| 149384C | -12 |  |  |  |  |  |  |  |  |
| 149385G | -11 |  |  |  |  |  |  |  |  |
| 149386C | -10 |  |  |  |  |  |  |  |  |
| 149387C | -9 |  |  |  |  |  |  |  |  |
| 149388G | -8 |  |  |  |  |  |  |  |  |
| 149389G | -7 |  |  |  |  |  |  |  |  |
| 149390G | -6 |  |  |  |  |  |  |  |  |
| 149391T | -5 |  |  |  |  |  |  |  |  |
| 149392T | -4 |  |  |  |  |  |  |  |  |
| 149393A | -3 |  |  | G |  |  |  |  |  |
| 149394T | -2 |  |  |  | G | A |  |  |  |
| 149395T | -1 |  |  |  |  |  |  |  | C |
| 149396G | 1 |  |  |  |  |  |  |  |  |
| 149397C | 2 |  |  |  |  |  |  |  |  |
| 149398T | 3 |  |  | C |  |  |  |  | A |
| 149399G | 4 |  |  |  |  |  | T |  |  |
| 149400A | 5 |  |  |  |  |  | G | G | T |
| 149401C | 6 |  |  |  |  |  |  |  |  |
| 149402A | 7 |  |  |  |  | G |  |  |  |
| 149403C | 8 |  |  |  | A |  |  |  |  |
| 149404G | 9 |  |  |  | C |  |  |  |  |
| 149405C | 10 |  |  |  |  |  |  |  | T |
| 149406T | 11 |  |  |  |  |  |  |  | C |
| 149407G | 12 |  |  |  |  | T |  | A |  |
| 149408T | 13 |  |  |  |  |  |  |  |  |
| 149409C | 14 |  |  |  |  |  |  |  |  |
| 149410C | 15 |  |  |  | A |  |  |  |  |
| 149411T | 16 |  |  |  |  |  |  |  |  |
| 149412C | 17 |  |  |  |  |  |  |  |  |
| 149413T | 18 |  |  |  |  |  | A, C |  |  |
| 149414G | 19 |  |  |  | A | C |  |  |  |
| 149415G | 20 |  |  |  |  |  |  |  |  |
| 149416C | 21 |  |  |  |  | A |  |  | 2-A |
| 149417G | 22 |  |  |  | A |  |  | A |  |
| 149418A | 23 |  | T |  |  | G |  | T |  |
| 149419C | 24 |  |  |  |  |  |  |  |  |
| 149420C | 25 |  |  |  |  |  |  |  |  |
| 149421T | 26 |  |  |  |  |  | C | C |  |
| 149422G | 27 |  |  |  |  |  |  | A |  |
| 149423T | 28 |  |  |  |  | C |  |  | C |
| 149424C | 29 |  |  |  |  |  |  |  |  |
| 149425G | 30 |  |  |  |  | T |  |  | 4A |
| 149426C | 31 |  |  |  |  |  |  |  |  |
| 149427T | 32 |  |  |  | C |  |  |  |  |
| 149428G | 33 |  | ins G |  |  |  |  | A, insG |  |
| 149429G | 34 |  |  |  |  |  |  |  |  |
| 149430A | 35 |  |  |  |  |  |  |  |  |
| 149431G | 36 |  |  |  |  |  |  |  |  |
| 149432A | 37 |  |  | G |  |  |  | ins G |  |
| 149433G | 38 |  |  |  |  |  |  |  |  |
| 149434G | 39 |  |  |  |  |  |  |  |  |
| 149435T | 40 |  |  |  |  |  |  |  |  |
| 149436T | 41 |  |  |  |  |  |  |  |  |
| 149437G | 42 |  |  |  |  |  |  |  |  |
| 149438G | 43 |  |  |  |  |  |  |  |  |
| 149439G | 44 |  |  |  |  |  |  | C |  |
| 149440C | 45 |  |  |  |  |  |  |  |  |
| 149441C | 46 |  |  |  |  |  |  |  |  |
| 149442T | 47 |  |  |  |  |  |  |  | A |
| 149443C | 48 |  |  |  |  |  |  |  |  |
| 149444C | 49 |  | 2 T |  |  | 3 T |  |  |  |
| 149445G | 50 |  | A | T |  |  |  |  |  |
| 149446G | 51 |  |  |  |  |  |  |  |  |
| 149447A | 52 |  |  |  |  | G | G |  | 5G |
| 149448T | 53 |  |  |  |  |  |  | C |  |
| 149449G | 54 |  |  |  |  |  |  |  |  |
| 149450C | 55 |  |  |  |  |  |  |  |  |
| 149451G | 56 |  |  |  |  |  |  |  |  |
| 149452C | 57 |  |  |  |  |  | T |  |  |
| 149453G | 58 |  |  |  |  |  |  |  |  |
| 149454C | 59 |  |  |  | T |  |  |  |  |
| 149455G | 60 |  |  |  |  |  |  | ins G |  |
| 149456G | 61 |  |  |  |  |  |  |  |  |
| 149457G | 62 |  |  |  |  |  |  |  |  |
| 149458G | 63 |  |  |  |  |  |  |  |  |
| 149459C | 64 |  |  |  |  |  |  |  |  |
| 149460T | 65 |  |  |  |  |  | C |  |  |
| 149461C | 66 |  |  |  |  |  |  |  |  |
| 149462T | 67 |  |  |  |  | C |  |  |  |
| 149463G | 68 |  |  |  |  |  |  |  |  |
| 149464G | 69 |  |  |  |  |  |  |  |  |
| 149465C | 70 |  |  |  |  |  |  |  | T |
| 149466C | 71 |  |  |  |  |  |  |  |  |
| 149467T | 72 |  | C |  |  |  |  |  |  |
| 149468A | 73 |  |  |  |  |  |  |  |  |
| 149469C | 74 |  |  |  |  |  |  |  |  |
| 149470C | 75 |  |  |  |  |  |  |  |  |
| 149471G | 76 |  |  |  |  |  |  |  | A |
| 149472G | 77 |  |  |  |  |  |  |  |  |
| 149473T | 78 |  |  |  |  |  |  |  |  |
| 149474G | 79 |  |  |  |  |  |  |  |  |
| 149475A | 80 |  | G |  | G |  |  | G |  |
| 149476C | 81 |  |  |  |  |  |  |  |  |
| 149477C | 82 |  |  |  | T |  |  |  |  |
| 149478C | 83 |  |  |  |  | T |  | A |  |
| 149479G | 84 |  |  |  |  |  |  |  |  |
| 149480G | 85 |  |  |  |  |  |  |  |  |
| 149481C | 86 |  |  |  |  |  |  |  |  |
| 149482T | 87 |  |  |  |  |  |  |  |  |
| 149483A | 88 |  |  |  |  |  |  | G |  |
| 149484G | 89 |  |  |  |  |  |  |  |  |
| 149485C | 90 |  |  |  |  |  |  |  |  |
| 149486C | 91 |  |  |  |  |  |  |  |  |
| 149487G | 92 |  |  |  |  |  |  |  |  |
| 149488G | 93 |  |  |  |  |  |  |  |  |
| 149489C | 94 |  |  |  |  |  |  |  |  |
| 149490C | 95 |  |  |  |  |  |  |  |  |
| 149491G | 96 |  |  |  |  | C |  |  |  |
| 149492C | 97 |  | T |  |  |  |  |  |  |
| 149493G | 98 |  |  | T | A |  |  | 2-A |  |
| 149494C | 99 |  | A |  |  |  |  |  |  |
| 149495T | 100 |  |  |  | C |  | C |  |  |
| 149496C | 101 |  |  |  |  |  |  |  |  |
| 149497C | 102 |  |  |  |  |  |  |  |  |
| 149498T | 103 |  |  |  |  |  |  |  |  |
| 149499G | 104 |  |  |  |  |  |  |  |  |
| 149500C | 105 |  |  |  |  |  |  |  |  |
| 149501T | 106 |  |  |  |  |  |  |  |  |
| 149502T | 107 |  |  |  |  |  |  | G | 2 C |
| 149503G | 108 |  |  |  |  |  |  |  |  |
| 149504A | 109 |  |  | G | G |  |  |  |  |
| 149505G | 110 |  |  |  |  |  |  |  | C |
| 149506C | 111 |  |  |  |  | T |  |  |  |
| 149507C | 112 |  |  |  |  |  |  |  |  |
| 149508G | 113 |  |  |  |  |  |  |  |  |
| 149509C | 114 |  |  |  |  |  |  |  |  |
| 149510C | 115 |  |  |  |  |  |  |  |  |
| 149511T | 116 |  |  |  |  |  |  |  |  |
| 149512G | 117 |  |  |  |  |  |  |  |  |
| 149513C | 118 |  |  |  |  |  |  |  |  |
| 149514C | 119 |  |  |  |  |  | G |  |  |
| 149515G | 120 |  |  | A |  |  |  | A |  |
| 149516G | 121 |  |  |  |  |  |  |  |  |
| 149517G | 122 |  |  |  |  |  |  |  |  |
| 149518G | 123 |  |  |  |  |  |  |  |  |
| 149519C | 124 |  |  | G |  |  |  |  |  |
| 149520C | 125 |  |  |  |  | T |  | ins G |  |
| 149521C | 126 |  |  |  |  |  |  |  | T |
| 149522G | 127 |  |  |  |  |  |  |  |  |
| 149523C | 128 |  |  |  |  |  |  |  |  |
| 149524G | 129 |  | A |  |  |  |  |  |  |
| 149525G | 130 |  |  |  |  |  |  |  |  |
| 149526G | 131 |  |  | C |  |  |  |  |  |
| 149527C | 132 |  |  |  |  |  |  |  |  |
| 149528C | 133 |  |  | T |  |  |  |  |  |
| 149529T | 134 |  |  |  | C |  |  |  |  |
| 149530G | 135 |  |  |  |  |  |  |  |  |
| 149531C | 136 |  |  |  | T |  |  |  |  |
| 149532T | 137 |  |  |  |  | C |  | C |  |
| 149533G | 138 |  |  |  |  |  |  |  |  |
| 149534T | 139 |  | 28 C | 7 C | 7 C |  | 3 C | 2 C | 4C |
| 149535T | 140 |  |  |  | del | C |  |  |  |
| 149536C | 141 |  | G |  | del |  |  |  |  |
| 149537T | 142 |  |  |  |  |  |  |  |  |
| 149538C | 143 |  |  |  |  |  |  |  |  |
| 149539T | 144 |  | 2 C | G |  |  | 3 C |  |  |
| 149540C | 145 |  |  |  | T |  |  |  |  |
| 149541G | 146 |  |  |  |  |  |  |  |  |
| 149542C | 147 |  |  |  |  |  | G |  |  |
| 149543G | 148 |  |  |  |  |  |  |  |  |
| 149544C | 149 |  |  |  |  |  |  |  |  |
| 149545G | 150 |  | A |  |  | A,T |  |  |  |
| 149546T | 151 |  |  |  | C |  |  |  |  |
| 149547C | 152 |  |  |  | 2 T |  |  |  |  |
| 149548C | 153 |  |  |  |  |  |  |  |  |
| 149549G | 154 |  |  |  |  |  |  |  |  |
| 149550A | 155 |  |  |  |  |  |  |  | 2 G |
| 149551G | 156 |  |  |  |  |  |  |  |  |
| 149552C | 157 |  |  | G |  | A |  |  |  |
| 149553G | 158 |  |  |  |  |  |  |  |  |
| 149554T | 159 |  |  |  |  |  |  |  |  |
| 149555C | 160 |  |  |  |  |  |  |  |  |
| 149556C | 161 |  |  | A |  |  |  |  |  |
| 149557C | 162 |  |  |  |  |  | T |  |  |
| 149558G | 163 |  |  |  |  |  |  |  |  |
| 149559A | 164 |  |  |  |  | 3 G |  | G | G |
| 149560C | 165 |  |  |  | T |  |  | G |  |
| 149561T | 166 |  |  |  |  | C |  |  |  |
| 149562C | 167 |  |  |  |  |  |  |  |  |
| 149563C | 168 |  |  |  |  |  |  |  |  |
| 149564C | 169 |  |  |  |  |  |  |  |  |
| 149565G | 170 |  |  |  |  |  | T |  |  |
| 149566G | 171 |  |  |  |  |  |  |  |  |
| 149567T | 172 |  |  | C |  |  |  | A |  |
| 149568G | 173 |  |  |  |  |  |  |  |  |
| 149569C | 174 |  |  |  |  |  |  |  |  |
| 149570C | 175 |  |  |  |  |  |  |  |  |
| 149571G | 176 |  |  |  |  |  |  |  |  |
| 149572G | 177 |  |  |  |  |  |  |  | A |
| 149573C | 178 |  |  |  |  |  |  |  |  |
| 149574C | 179 |  |  |  | T |  |  |  |  |
| 149575C | 180 |  |  | 3 T |  |  |  |  |  |
| 149576G | 181 |  | 2 ins G |  | C | A |  | ins G |  |
| 149577G | 182 |  |  |  |  |  |  |  |  |
| 149578G | 183 |  |  |  |  |  |  |  |  |
| 149579T | 184 |  | C |  |  |  |  |  |  |
| 149580C | 185 |  |  |  |  |  |  |  |  |
| 149581C | 186 |  | T |  | A |  |  |  |  |
| 149582G | 187 |  |  |  |  | T | C |  |  |
| 149583G | 188 |  | A |  |  |  |  |  |  |
| 149584G | 189 |  | T | T |  |  |  |  | T |
| 149585T | 190 |  |  |  |  |  | C |  |  |
| 149586C | 191 |  |  |  |  |  |  | T |  |
| 149587T | 192 |  |  |  |  |  |  |  |  |
| 149588C | 193 |  |  | T |  | A |  |  |  |
| 149589T | 194 |  |  |  |  |  |  |  |  |
| 149590G | 195 |  |  | T |  | A |  |  | A |
| 149591A | 196 |  |  |  |  | G |  |  |  |
| 149592C | 197 |  |  |  |  |  |  |  |  |
| 149593C | 198 |  |  |  |  |  |  |  |  |
| 149594C | 199 |  |  |  |  |  |  |  |  |
| 149595A | 200 |  |  | C |  |  |  | G |  |
| 149596C | 201 |  |  |  |  |  |  |  |  |
| 149597C | 202 |  |  |  |  |  |  |  |  |
| 149598C | 203 |  |  |  |  |  |  |  |  |
| 149599G | 204 |  |  |  |  |  |  | del |  |
| 149600G | 205 |  |  |  |  |  | ins G | T | ins G |
| 149601G | 206 |  |  |  |  |  |  | A |  |
| 149602G | 207 |  |  |  | 9A |  |  |  |  |
| 149603G | 208 |  |  |  |  |  |  |  |  |
| 149604C | 209 |  |  |  |  |  |  |  |  |
| 149605G | 210 |  |  |  |  |  |  |  | ins G |
| 149606G | 211 |  |  |  |  |  | C |  |  |
| 149607C | 212 |  |  |  |  |  |  | ins C |  |
| 149608G | 213 |  |  |  | 2 C | del |  | A ,C |  |
| 149609G | 214 |  |  |  |  |  |  |  |  |
| 149610G | 215 |  |  |  |  | A, T |  |  |  |
| 149611G | 216 |  |  |  |  |  |  |  |  |
| 149612A | 217 |  | ins A |  |  | G |  |  |  |
| 149613A | 218 |  |  |  |  |  |  |  |  |
| 149614G | 219 |  |  |  |  | A |  |  | A |
| 149615G | 220 |  |  |  |  |  |  | C |  |
| 149616C | 221 |  |  |  |  | G |  | G |  |
| 149617G | 222 |  |  |  |  | C |  |  |  |
| 149618G | 223 |  |  |  |  | A |  |  |  |
| 149619C | 224 |  |  |  |  | G, G |  |  |  |
| 149620G | 225 |  | A | A | 3A |  |  | A, C |  |
| 149621A | 226 |  |  |  |  |  | G |  |  |
| 149622G | 227 |  |  |  |  |  |  |  |  |
| 149623G | 228 |  |  |  |  |  |  |  |  |
| 149624G | 229 |  |  |  |  |  |  |  |  |
| 149625C | 230 |  |  |  |  |  |  |  |  |
| 149626C | 231 |  |  |  |  |  |  |  |  |
| 149627A | 232 |  |  |  |  |  |  |  |  |
| 149628C | 233 |  |  |  |  |  |  |  |  |
| 149629C | 234 |  |  |  |  | G, T |  |  |  |
| 149630G | 235 |  |  |  |  |  |  |  |  |
| 149631T | 236 |  |  |  |  |  |  |  |  |
| 149632G | 237 |  |  |  |  |  |  |  |  |
| 149633C | 238 |  |  |  | del |  |  |  |  |
| 149634C | 239 |  | T |  |  |  |  |  |  |
| 149635C | 240 |  |  |  |  | A |  |  |  |
| 149636C | 241 |  |  |  |  |  |  |  |  |
| 149637C | 242 |  |  |  |  | G |  |  |  |
| 149638G | 243 |  |  |  |  |  |  |  |  |
| 149639T | 244 |  |  |  |  |  |  |  |  |
| 149640G | 245 |  |  |  |  |  |  |  |  |
| 149641C | 246 |  | T |  |  |  |  |  |  |
| 149642G | 247 |  |  |  |  | A |  |  |  |
| 149643C | 248 |  |  |  |  |  |  |  |  |
| 149644T | 249 |  |  |  |  |  |  |  | A |
| 149645C | 250 |  |  |  |  | A |  |  |  |
| 149646T | 251 |  |  |  |  |  |  |  |  |
| 149647C | 252 |  |  |  |  | G |  |  |  |
| 149648C | 253 |  |  |  |  |  |  |  |  |
| 149649G | 254 |  |  |  |  |  |  |  |  |
| 149650C | 255 |  |  |  |  |  |  |  |  |
| 149651T | 256 |  | C |  |  |  |  |  |  |
| 149652G | 257 |  |  |  |  |  |  |  |  |
| 149653C | 258 |  |  |  |  |  |  |  |  |
| 149654G | 259 |  |  |  |  |  | A |  |  |
| 149655G | 260 |  |  |  |  |  |  |  |  |
| 149656G | 261 |  |  |  |  |  |  |  |  |
| 149657C | 262 |  |  |  |  | T |  |  |  |
| 149658G | 263 |  |  |  |  |  |  |  |  |
| 149659C | 264 |  |  |  |  |  |  |  |  |
| 149660C | 265 |  |  |  |  |  |  |  |  |
| 149661C | 266 |  |  |  |  |  |  |  |  |
| 149662G | 267 |  |  |  |  |  |  |  |  |
| 149663G | 268 |  |  |  |  |  |  |  |  |
| 149664G | 269 |  |  |  |  |  |  |  |  |
| 149665G | 270 |  |  |  |  |  |  |  |  |
| 149666C | 271 |  | T |  |  |  |  |  |  |
| 149667G | 272 |  |  |  |  |  |  |  |  |
| 149668G | 273 |  |  |  |  |  |  |  |  |
| 149669C | 274 |  |  |  |  | del |  |  |  |
| 149670C | 275 |  |  |  |  |  |  |  |  |
| 149671G | 276 |  |  |  |  |  |  |  |  |
| 149672C | 277 |  |  |  |  |  |  |  |  |
| 149673G | 278 |  | A |  |  | 2-A |  |  |  |
| 149674A | 279 |  |  |  |  |  |  |  |  |
| 149675C | 280 |  |  |  |  |  |  |  |  |
| 149676A | 281 |  |  |  |  |  |  |  |  |
| 149677A | 282 |  |  |  |  |  | G |  |  |
| 149678C | 283 |  |  |  |  |  |  |  |  |
| 149679C | 284 |  |  |  |  |  |  |  |  |
| 149680C | 285 |  |  |  |  | A |  |  |  |
| 149681C | 286 |  |  |  |  |  |  |  |  |
| 149682A | 287 |  |  |  | T | G |  |  | 3T |
| 149683C | 288 |  |  |  |  |  |  |  |  |
| 149684C | 289 |  |  |  |  |  |  |  |  |
| 149685C | 290 |  |  |  |  | T | 3 G | 2 G |  |
| 149686C | 291 |  |  |  |  |  |  |  | 3T |
| 149687G | 292 |  |  |  |  | T |  |  |  |
| 149688C | 293 |  |  |  |  |  |  |  |  |
| 149689T | 294 |  |  |  |  |  |  |  | G |
| 149690G | 295 |  |  |  | ins G |  |  |  | A |
| 149691G | 296 |  |  |  |  |  | A |  |  |
| 149692C | 297 |  |  |  |  |  |  |  |  |
| 149693T | 298 |  |  | 2 C |  | A |  |  |  |
| 149694C | 299 |  |  |  |  |  | T |  |  |
| 149695C | 300 |  |  |  |  |  |  |  |  |
| 149696G | 301 |  |  |  | C |  |  |  |  |
| 149697T | 302 |  |  |  |  | C |  |  |  |
| 149698G | 303 |  |  |  |  | A |  |  |  |
| 149699C | 304 |  |  |  |  |  |  |  |  |
| 149700C | 305 |  |  |  |  |  | T |  |  |
| 149701G | 306 |  |  |  |  |  |  |  |  |

^a^Nucleotide position number is in reference to GenBank AL592188.

^b^Site is relative to *rRNA* transcription start.
